# Common and Distant Structural Characteristics of Feruloyl Esterase Families from *Aspergillus oryzae*

**DOI:** 10.1371/journal.pone.0039473

**Published:** 2012-06-22

**Authors:** D. B. R. K. Gupta Udatha, Valeria Mapelli, Gianni Panagiotou, Lisbeth Olsson

**Affiliations:** 1 Department of Chemical and Biological Engineering, Industrial Biotechnology, Chalmers University of Technology, Gothenburg, Sweden; 2 Center for Biological Sequence Analysis, Department of Systems Biology, Technical University of Denmark, Lyngby, Denmark; 3 Novo Nordisk Foundation Center for Biosustainability, Technical University of Denmark, Hørsholm, Denmark; University of Cincinnati College of Medicine, United States of America

## Abstract

**Background:**

Feruloyl esterases (FAEs) are important biomass degrading accessory enzymes due to their capability of cleaving the ester links between hemicellulose and pectin to aromatic compounds of lignin, thus enhancing the accessibility of plant tissues to cellulolytic and hemicellulolytic enzymes. FAEs have gained increased attention in the area of biocatalytic transformations for the synthesis of value added compounds with medicinal and nutritional applications. Following the increasing attention on these enzymes, a novel descriptor based classification system has been proposed for FAEs resulting into 12 distinct families and pharmacophore models for three FAE sub-families have been developed.

**Methodology/Principal Findings:**

The feruloylome of *Aspergillus oryzae* contains 13 predicted FAEs belonging to six sub-families based on our recently developed descriptor-based classification system. The three-dimensional structures of the 13 FAEs were modeled for structural analysis of the feruloylome. The three genes coding for three enzymes, viz., A.O.2, A.O.8 and A.O.10 from the feruloylome of *A. oryzae,* representing sub-families with unknown functional features, were heterologously expressed in *Pichia pastoris,* characterized for substrate specificity and structural characterization through CD spectroscopy. Common feature-based pharamacophore models were developed according to substrate specificity characteristics of the three enzymes. The active site residues were identified for the three expressed FAEs by determining the titration curves of amino acid residues as a function of the pH by applying molecular simulations.

**Conclusions/Significance:**

Our findings on the structure-function relationships and substrate specificity of the FAEs of *A. oryzae* will be instrumental for further understanding of the FAE families in the novel classification system. The developed pharmacophore models could be applied for virtual screening of compound databases for short listing the putative substrates prior to docking studies or for post-processing docking results to remove false positives. Our study exemplifies how computational predictions can complement to the information obtained through experimental methods.

## Introduction

Feruloyl esterases (FAEs) have gained importance in biofuel, medicine and food industries due to their capability of hydrolyzing carbohydrate esters in wood polymers and synthesizing high added-value molecules through esterification and transesterification reactions [1]–[3]. Even though FAEs possess a common characteristic feature of the Ser-His-Asp triad forming the active site, variations in amino acid sequences forming surface loops and additional domains allow them to accommodate different aromatic substrates. By using the sequence properties of FAEs, Udatha *et al.* [4] proposed a new classification system with common functional characteristics for each of the 12 predicted FAE families. Briefly, FAE-related sequences of fungal, bacterial and plant origin have been collected and clustered using self-organizing maps followed by k-means clustering into distinct groups based on amino acid composition and physico-chemical composition descriptors derived from the respective amino acid sequence. A Support Vector Machine (SVM) model has been developed for the classification of new FAEs into the pre-assigned clusters. Pharmacophore models for the new FAE families have been designed for which sufficient information of known substrates existed. The development of pharmacophore models based on substrate spectra for specific FAE sub-families is expected to be a crucial tool for designing the application of members of the particular sub-family to completely novel and targeted substrates. Virtual screening with the developed pharmacophores of chemical and natural compound databases could reveal unique opportunities for FAEs-based-biocatalysis for synthesis of compounds with altered or improved bioactive properties. However, a detailed understanding of the structural factors responsible for stability and activity of FAE families is still not available.

In the present work we predicted that *Aspergillus oryzae* possesses thirteen FAEs that fall into six different FAE sub-families (Table 1), and we collectively designated them as the ‘feruloylome’. Out of the 13 members, three FAEs have been partially characterized [5], [6]. The structural characterization of these enzymes and the connection with their functionality remains to be mapped. Although significant partial characterization experimental work has been reported on FAEs [1], [5], [7]–[11], computational studies to understand FAE structural characteristics have not emerged yet. Recent work has demonstrated that various structural bioinformatics tools such as homology modeling and molecular dynamics simulations can provide information to gain insights of protein/receptor functional elements [12]–[16]. Due to absence of crystal structures for the *A. oryzae* feruloylome members, we exploited here a combined approach of molecular modeling and molecular dynamics simulations to investigate the structural features of FAEs. Identifying the residues involved in the catalytic mechanism and understanding the environment of the enzyme binding pocket would expand our toolbox for targeted engineering. Furthermore, we expressed and investigated the structure-function relationships of three feruloylome members by collecting experimental data determining the substrate specificity towards 15 substrates and enzyme activity as a function of pH using Circular Dichroism (CD) spectroscopy.

**Table 1:** Feruloylome members of *Aspergillus oryzae.*

ID^1^	GI number and Protein accession	Amino acid length	Sub-family^2^
A.O.1	gi|83776083|dbj|BAE66202.1|	446	FEF 4A
A.O.2	gi|83768497|dbj|BAE58634.1|	573	FEF 4A
A.O.3	gi|83770997|dbj|BAE61129.1|	525	FEF 4B
A.O.4	gi|169768564|ref|XP_001818752.1|	281	FEF 6B
A.O.5	gi|169783734|ref|XP_001826329.1|	307	FEF 6B
A.O.6	gi|83776294|dbj|BAE66413.1|	516	FEF 7A
A.O.7	gi|83775577|dbj|BAE65697.1|	514	FEF 7A
A.O.8	gi|83771599|dbj|BAE61730.1|	524	FEF 7A
A.O.9	gi|83769703|dbj|BAE59838.1|	502	FEF 7A
A.O.10	gi|83775432|dbj|BAE65552.1|	588	FEF 12B
A.O.11	gi|83766949|dbj|BAE57089.1|	526	FEF 4B
A.O.12	gi|83769004|dbj|BAE59141.1|	529	FEF 11A
A.O.13	gi|83766486|dbj|BAE56626.1|	540	FEF 12B

1 For convenience the ID designations were used throughout the article.

2 According to descriptor based FAE classification system [4].

## Methods

### Sequences

Nucleotide and amino acid sequence information were retrieved from the National Center for Biotechnology Information [17]. The gene sequences, mRNA sequences and amino acid sequences for the feruloylome of *Aspergillus oryzae* are available in the Supporting Information S1.

**Figure 1:**
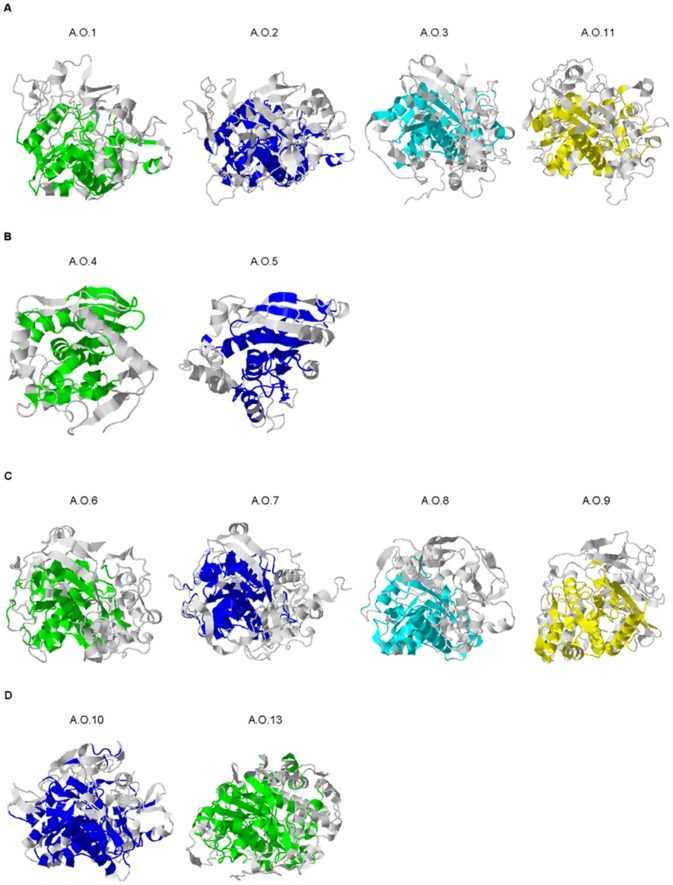
Regions of similar SSEs among the FEF family members shown as ribbon structures. Structurally similar regions are colored whereas dissimilar regions are depicted in grey. (A) Structural similarity in the secondary structure elements of FEF 4 family members. (B) Structural similarity in the secondary structure elements of FEF 6 family members. (C) Structural similarity in the secondary structure elements of FEF 7 family members. (D) Structural similarity in the secondary structure elements of FEF 12 family members.

### *In Silico* Analysis

Primary structure analysis of the FAE amino acid sequences was performed using ProtParam, by computing various physical and chemical parameters including the molecular weight, theoretical pI, extinction coefficient and absorbance for a user entered sequence [18]. The amino acid sequences were analyzed for the presence of native signal peptide using the SignalP 3.0 server, which predicts the presence and location of signal peptide cleavage sites in amino acid sequences based on a combination of several artificial neural networks and hidden Markov models [19]–[21]. The summary of calculated physico-chemical parameters and post-translational modification analysis of the mature FAEs are given in Table S1. Three-dimensional atomic models for the *A. oryzae* feruloylome sequences were modeled from multiple threading alignments [22] and iterative structural assembly simulations using I-TASSER algorithm [23]–[26]. Structure refinement of modeled structures was carried out using the Discovery Studio software suite version 3.0 (Accelrys Inc, USA). The Prepare Protein protocol package in Discovery Studio suite was used for inserting missing atoms in incomplete residues, modeling missing loop regions [27], deleting alternate conformations (disorder), standardizing atom names, and protonating titratable residues using predicted pKs [28]. The Side-Chain Refinement protocol was used for each structure to optimize the protein side-chain conformation based on systematic searching of side-chain conformation and CHARMM Polar H energy minimization [29] using the ChiRotor algorithm [30]. Smart Minimizer algorithm was used for the minimization process which performs 1000 steps of Steepest Descent with a RMS gradient tolerance of 3, followed by Conjugate Gradient minimization for faster convergence towards a local minimum [31]. Structure evaluations were carried out using DOPE, which is an atomic based statistical potential in MODELER package for model evaluation and structure prediction [32]. Structure verifications were carried out using VerifyProtein-Profiles-3D that allows evaluating the fitness of a protein sequence in its current 3D environment [33].

### Gene Cloning, Expression and Purification

#### Genes and vectors

The RNA isolated from *Aspergillus oryzae* as described earlier [34] was a kind gift from Department of Systems Biology-DTU. DNase treatment of the RNA sample and further RNA cleanup were performed according to the manufacturer’s instructions using RNase-Free DNase Set and RNeasy Mini Kit, respectively (QIAGEN Nordic, Sweden). Quality and quantity of the RNA samples were determined by using a BioPhotometer (Eppendorf, Germany). The purified RNA was stored at −80°C until further processing. The primers were designed to amplify the mature-protein coding sequences excluding the signal peptide. cDNA synthesis was carried out using the Transcriptor One-Step RT-PCR Enzyme Mix (Roche Diagnostics GmbH, Germany). Purification of the cDNA from the RT-PCR product mixture was performed using the QIAquick PCR Purification Kit according to the manufacturer’s protocol (QIAGEN Nordic, Sweden). Primers were designed using the OligoCalc tool [35] and Clone Manager software version 9 (Scientific & Educational Software, USA). The list of oligo nucleotide primers used is given in the Table S2. The plasmid pPICZα-C vector (Invitrogen, UK) was used for cloning and expression in *Pichia pastoris*.

#### Cloning

Restriction of purified cDNA from Transcriptor One-Step RT-PCR and restriction of pPICZα-C vector were carried out with respective restriction enzymes (Fermentas, Lithuania) reported in Table S2. Purification of restricted DNA molecules was performed using the QIAquick PCR Purification Kit (QIAGEN Nordic, Sweden). The genes were cloned into the pPICZα-C vector (Invitrogen, UK), which includes the α-factor signal sequence for secretion of proteins, c-*myc* epitope and 6-His tag to the c-terminal of each recombinant protein. Ligation of respective restricted vectors and restricted inserts was carried out using T4 DNA Ligase according to the manufacturer’s protocol (Fermentas, Lithuania). Isolation of plasmid constructs from transformed *E. coli* cells was carried out using GenElute HP plasmid miniprep kit (SIGMA-ALDRICH, USA) and confirmation of the clones was done by restriction analysis. Correct insertion of the FAE-encoding genes and absence of mutations were checked by DNA sequencing (Eurofins MWG operon, Germany).

#### Media and strains

The *Escherichia coli* strain TOP10 (Invitrogen, UK) was used as intermediate host for cloning and plasmid amplification and was grown on LB-medium [36] under appropriate selective conditions (zeocin 25 mg·L^−1^). For expression of recombinant proteins the protease-deficient *P. pastoris* strain SMD1168H (Invitrogen, UK) was transformed with pPICZα-C/FAE constructs and positive clones were selected on selective YPDS medium (Sorbitol 182.2 g·L^−1^; yeast extract 10 g·L^−1^; peptone 20 g·L^−1^; dextrose 10 g·L^−1^; 25 mg·L^−1^ zeocin). Expression experiments were carried out in Buffered Glycerol-Complex Medium (BMGY) and Buffered Methanol-Complex Medium (BMMY) during induction phase. The complex expression media, prepared in 100 mM potassium phosphate buffer (pH 6.0), contained 1% w/v yeast extract, 2% w/v peptone, 1.34% w/v Yeast Nitrogen Base, 4×10^−5^% w/v biotin, 1% v/v glycerol and 1% v/v methanol in case of BMGY and BMMY, respectively.

**Figure 2:**
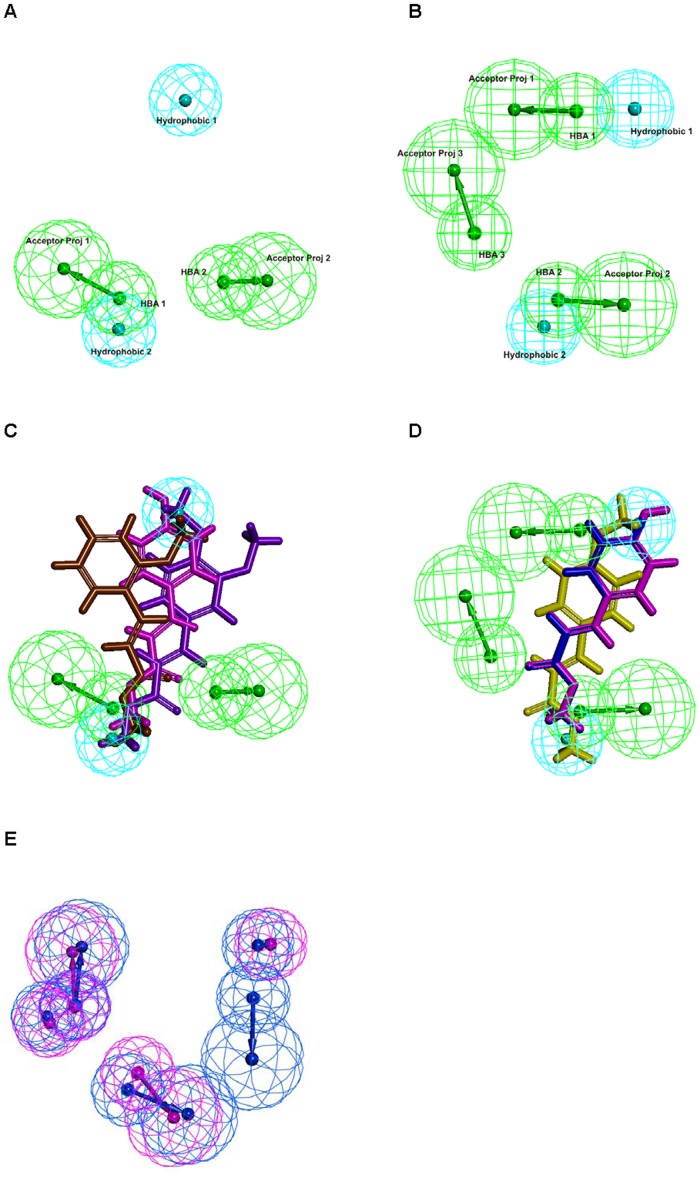
Common feature pharmacophore models for FEF 7A and FEF 12B sub-families. (A) Pharmacophore model features of A.O.8 enzyme. (B) Pharmacophore model features of A.O.10 enzyme. (C) Alignment of inactive substrates of A.O.8 against its pharamacophore model. MPC, M2M and M34DC substrates are shown in magenta, brown and purple stick models, respectively. (D) Alignment of inactive substrates of A.O.10 against its pharamacophore model. MPC, MC and M4M substrates are shown in magenta, blue and yellow stick models, respectively. (E) Comparison of A.O.8 and A.O.10 pharmacophore models showing the addition feature HBA1 attained by A.O.10 inherited from its different substrate spectra.

**Figure 3:**
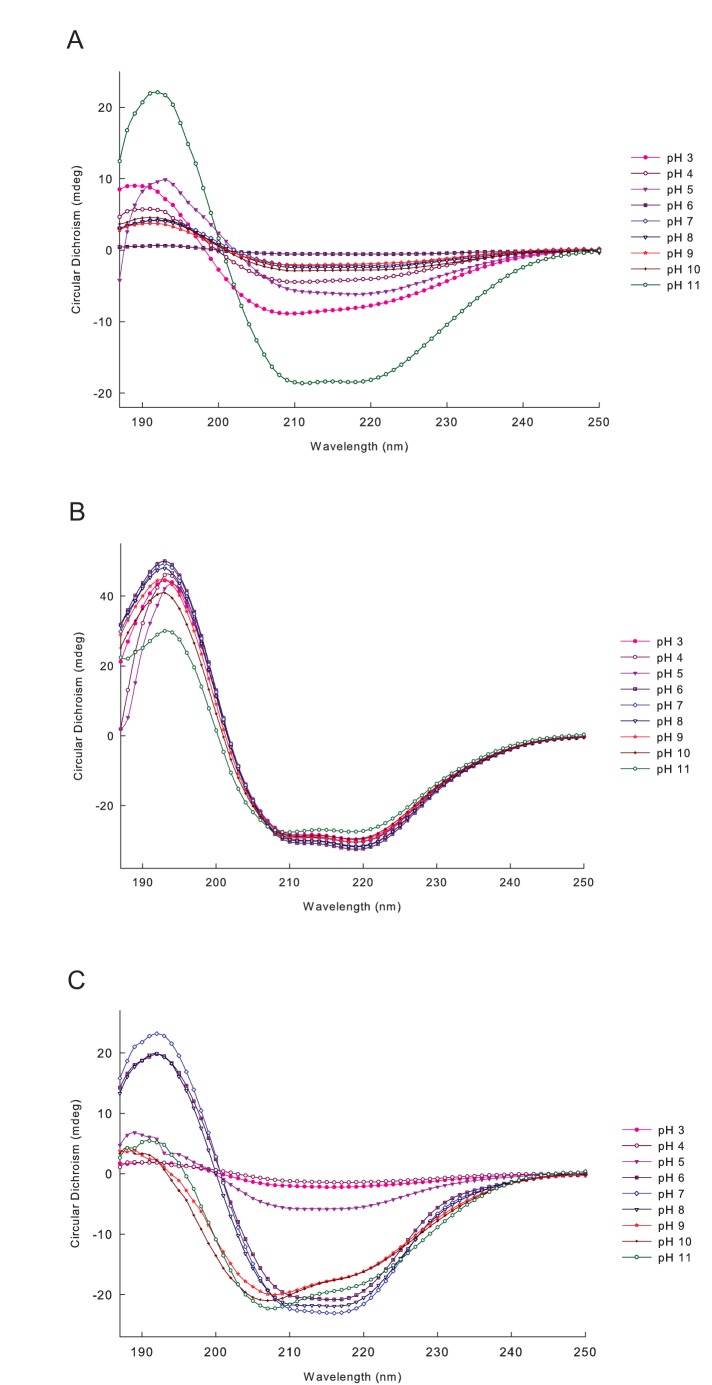
Circular dichroism spectroscopy profile for the three expressed FAEs. (*A*) CD spectra at different pH for A.O.2 enzyme. (*B*) CD spectra at different pH for A.O.8 enzyme. (*C*) CD spectra at different pH for A.O.10 enzyme.

**Figure 4:**
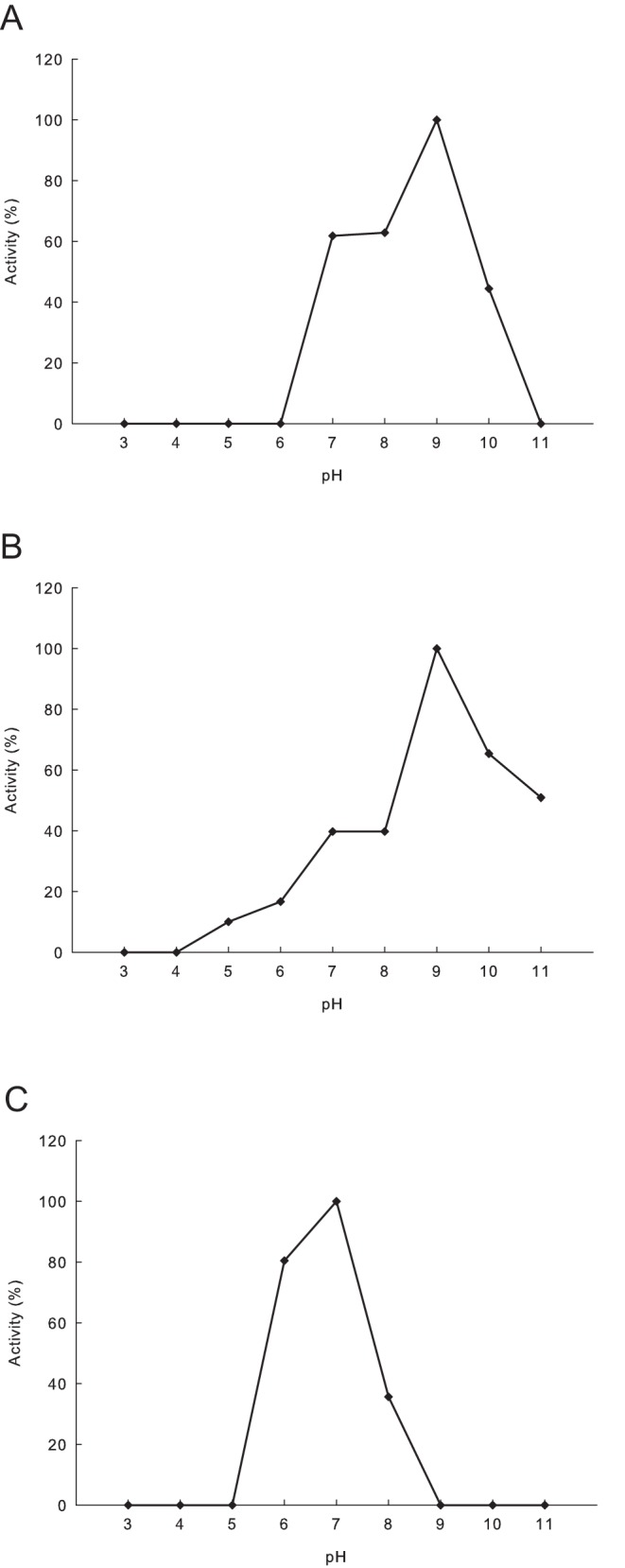
pH dependent relative activity profiles for the three expressed FAEs. Values are expressed relative to the hydrolytic efficiency of respective FAE for methyl ferulate. (A) Effect of pH on A.O.2 activity. (B) Effect of pH on A.O.8 activity. (C) Effect of pH on A.O.10 activity.

#### Transformation of *P. pastoris*

To promote the integration of the pPICZα-C/FAE constructs in the genome of *P. pastoris*, respective constructs were linearized using the *PmeI* restriction enzyme followed by purification of the linearized constructs prior to transformation. Transformations of *P. pastoris* with respective constructs were carried out by electroporation according to the manufacturer’s protocol (Invitrogen, UK). To test integration of respective vector construct into the genome, direct PCR Screening of 12 *Pichia clones* for each type of insert was performed using two sets of primers. The first set contains 5'AOX1 primer and 3'primer (Table S2) of the respective insert; the second set contains 3'AOX1 primer and 5'primer (Table S2) of the respective insert. The PCR products obtained from respective primer sets were analyzed on agarose gel.

#### Screening of colonies for highly expressing *P. pastoris* transformants

Six positive colonies of each transformation were picked and small-scale expression trials were performed. Respective transformants were pre-cultured overnight in 50 mL BMGY medium (in 500 ml baffled flasks) at 30°C and 220 rpm overnight. Cells were harvested and diluted in 150 mL BMMY medium to a final OD_600_ of 1.0. Cultures were grown in 1L baffled flasks at 30°C and 220 rpm for 96 hours and methanol was added to a concentration of 0.5% every 24 hours. Samples were taken periodically and the supernatants were analyzed for FAE activity in the extracellular medium.

#### Purification of recombinant feruloyl esterase

After 96 hours of incubation, respective liquid cultures of *P. pastoris* were filtered using 0.2 µm bottle-top vacuum filters (Nalge Nunc International, USA). The filtrate was pre-equilibrated with 20 mM Imidazole and loaded onto Ni Sepharose™ 6 Fast Flow resin (GE Healthcare, USA), which was pre-equilibrated with buffer I (20 mM sodium phosphate buffer, 500 mM NaCl, pH 7.4). The resin was washed with buffer II (20 mM sodium phosphate buffer, 500 mM NaCl, 20 mM Imidazole, pH 7.4), and the target protein was eluted with buffer III (20 mM sodium phosphate buffer, 500 mM NaCl, 500 mM Imidazole, pH 7.5). Buffer exchange was performed using a 10 kDa Amicon^R^ Ultra (Millipore, Ireland) centrifugal filters to remove the imidazole. The protein concentration was determined by the Bradford method using bovine serum albumin as a standard [37]. Molecular weight of expressed FAEs was checked by SDS-PAGE using TGX precast gels of 4–15% resolving gel (Bio-Rad, USA). Protein bands were visualized by SYPRO ruby protein gel stain (Invitrogen, UK).

### Enzyme Activity Assay

The assay was based on a previously reported spectrophotometric method for determination of FAE activity recommended by Biocatalysts Limited, Wales, UK [38], [39]. The absorption spectra (FLUOstar Omega, BMG LABTECH, Germany) of the methyl esters of cinnamic acids and their hydrolysis products was monitored at 240–600 nm and their absorption maxima were determined [40]. FAE activity was expressed in milliUnits (mU); 1 mU was equal to 1 nmol of ferulic acid or respective cinnamic acid released in 1 ml of the reaction medium after 1 min of incubation [38]. The assays were done in triplicates and *K_m_* values were calculated using SigmaPlot for Windows, Version 12.0 (Systat Software Inc, USA).

The expressed recombinant FAEs were tested against 15 methyl cinnamate esters (obtained from Aapin Chemicals, UK) viz., Methyl ferulate or Methyl 4-hydroxy-3-methoxy cinnamate (MFA), Methyl caffeate or Methyl 3,4-dihydroxy cinnamate (MCA), Methyl p-coumarate or Methyl 4-hydroxy cinnamate (MPC), Methyl sinapate or Methyl 4-hydroxy-3,5-dimethoxy cinnamate (MSA), Methyl 2-hydroxy cinnamate (M2C), Methyl 3-hydroxy cinnamate (M3C), Methyl cinnamate (MC), Methyl 3,4,5-trimethoxy cinnamate (MTM), Methyl 2-methoxy cinnamate (M2M), Methyl 3-methoxy cinnamate (M3M), Methyl 4-methoxy cinnamate (M4M), Methyl 3,4-dimethoxy cinnamate (M34DC), Methyl 3,5-dimethoxy cinnamate (M35DC), Methyl 3-hydroxy-4-methoxy cinnamate (M34MC) and Methyl 4-hydroxy-3-methoxy phenyl propionate (M43PP).

### Circular Dichroism Spectroscopy

The far-UV Circular Dichroism (CD) measurements were carried on a Chirascan™ CD Spectrometer equipped with a thermostated cell holder (Applied Photophysics Limited, UK). Chirascan™ CD Spectrometer is endowed with photon flux for a 1 nm bandwidth in excess of 10^13^ per second for all UV wavelengths from 360 nm to 180 nm. During CD spectroscopy analysis, respective purified 6×His N-terminally tagged recombinant proteins (0.5 mg/mL) were resuspended in the corresponding buffer (pH 3.0–pH 11.0) and analysed at room temperature (∼25°C). UV CD spectra between 185 and 250 nm were collected with a data pitch of 0.1 nm, bandwidth of 2.0 nm and scanning speed of 50 nm/min [41]. Each sample was measured in triplicate, and data between 190–240 nm were analyzed using the K2D2 method. K2D2 method uses a self-organized map of spectra from proteins with known structure to deduce a map of protein secondary structure that is used to develop the predictions [42]–[44].

### Molecular Simulations and Amino Acid Titration Curves

CHARMM (Chemistry at HARvard Molecular Mechanics) was used to perform molecular simulations. Calculations of protein ionization and residue pK to obtain amino acid titration curves in the present study were performed using an implementation of the Generalized Born solvation model in CHARMM and the atomic parameters were taken from either CHARMM or CHARMM polar hydrogen force fields (Discovery Studio 3.0, Accelrys Inc, USA). The side-chains of certain amino acid residues viz., Asp, Glu, Arg, Lys, His, Tyr, Cys, and the N-termini and C-termini of each protein chain, are titratable. Using the theory developed by Bashford and Karplus [45], the pK of a titratable residue in a protein structure can be predicted from the pK of the model compounds based on the electrostatic interactions of this residue to the other residues.

**Figure 5:**
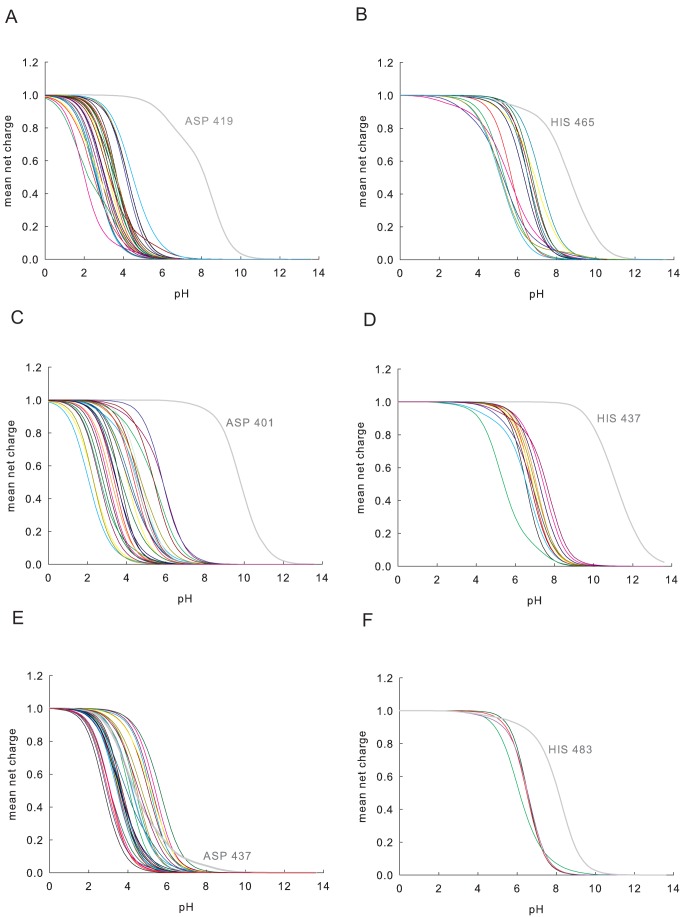
Titration curves of aspartic acid and histidine amino acid residues as a function of pH obtained by molecular simulations. (A) Protonation profiles of aspartic acid residues in A.O.2 protein as a function of pH. The curve for ASP 419 is shown in grey. The colored curves correspond to titration curves of all other aspartic acid residues present in the protein. (B) Protonation profiles of histidine residues in A.O.2 protein as a function of pH. The curve for HIS 465 is shown in grey. The colored curves correspond to titration curves of all other histidine residues present in the protein. (C) Protonation profiles of aspartic acid residues in A.O.8 protein as a function of pH. The curve for ASP 401 is shown in grey. (D) Protonation profiles of histidine residues in A.O.8 protein as a function of pH. The curve for HIS 437 is shown in grey. (E) Protonation profiles of aspartic acid residues in A.O.10 protein as a function of pH. The curve for ASP 437 is shown in grey. (F) Protonation profiles of histidine residues in A.O.10 protein as a function of pH. The curve for HIS 483 is shown in grey.

## Results and Discussion

### Protein Model Structures of the *A. oryzae* Feruloylome

Our effort in finding structure-function relationships in FAEs requires three-dimensional protein structures, hence several homology-model structures were derived each having a C-score. C-score is a confidence score that gives an estimate of the quality of predicted models and is calculated based on the significance of threading template alignments and the convergence parameters of the structure assembly simulations. C-score is typically in the range of (−5,2), where a C-score of higher value signifies a model with a high confidence and vice-versa [23], [24], [26]. All modeled structures of *A. oryzae* feruloylome were in the specified C-score range. The Protein Data Bank (PDB) structures used for multi template modeling and their respective secondary structure alignments are given in the Supporting Information S2. The coordinates of the model structures with high C-scores were submitted to the Protein Model DataBase (PMBD) [46]. The solvent surface rendering (probe radius of 1.4) of the modeled structures colored according to the secondary structure elements and respective PMDB accession codes is given in Figure S1. The verification scores viz., DOPE (Discrete Optimized Protein Energy) score, DOPE-HR score, Verify score and the potential energies of the modeled structures (see Table S3) revealed high quality of the models.

The DOPE score of a protein model is related to the conformational energy which measures the relative stability of a conformation with respect to other possible conformations of the same protein [32]. A lower DOPE score indicates smaller model errors which is the case for all the modeled FAEs in the present study (Table S3). The DOPE score is not normalized with the protein size, so DOPE scores of proteins with different sequences should not be compared directly.

The Verify Protein protocol is generally employed in the final phase of a homology modeling project or for testing a preliminary protein structure based on experimental data. The Verify Protein score (Profiles-3D) allows to evaluate the fitness of a protein sequence in its current 3D environment [47]. The Verify scores for the modeled FAEs are shown in Table S3 along with the expected high and low Verify scores for proteins of similar size. Verify scores close to the expected high value are positive indicators showing the quality of our modeled FAE structures.

Finally, the CHARMM based Energy minimization of the modeled structures was performed to remove steric overlaps that cause bad contacts; the initial potential energies of starting structures and the potential energy of respective minimized structures are given in Table S3. This high quality refined model structures are the ones we used for analysis of conserved structural elements and probing of the binding pockets.

### Structural Promiscuity of the FAE Families with Conserved Architecture within Each Family

Enzyme promiscuity can be attained through variations in conformation around the binding pocket. However, the same configurational environment and essential structural features can also be preserved for promiscuous activity or substrate profiles [48]–[53]. Three-dimensional structure alignments of the feruloylome of *A. oryzae* using PDBeFold [54]–[58] revealed the structural elements that are common within FAE families and that can potentially be related to substrate specificities. The cross-structure statistics for 3D structure alignment of the 13 FAEs are given in Table 2. The proteins within a FAE family show significant structural similarity especially in the secondary structural elements surrounding the binding pocket. The FAEs A.O.1, A.O.2, A.O.3 and A.O.11 which we predicted (based on the sequence descriptors) [4] as members of the feruloyl esterase family (FEF) 4 showed structural similarity with an overall RMSD of 3.3 Å over 187 structurally aligned residues. The FAEs A.O.4 and A.O.5, which we predicted as members of the FEF 6, showed structural similarity with an overall RMSD of 2.7 Å over 134 structurally aligned residues. The FAEs A.O.6, A.O.7, A.O.8 and A.O.9, which we predicted as members of the FEF 7, showed structural similarity with an overall RMSD of 3.1 Å over 192 structurally aligned residues. In addition, the FAEs A.O.10 and A.O.13, predicted as FEF 12 members, showed structural homology with an overall RMSD of 2.9 Å over 262 structurally aligned residues. The predicted amino acid sequence of A.O.10 shows complete identity with the amino acid sequence of BAA09656, which has been identified as tannase [59]. Based on the fact that majority of FAE and FAE related sequences in descriptor based classification system belong to tannase and feruloyl esterase protein superfamily [4], it is not surprising that A.O.10 has been identified and classified as tannase in the past [59]. Taking into consideration the number of amino acid residues of each protein, approximately 50% of the residues were structurally aligned with its family member proteins, but it should not be overlooked that the sequence homology is still quite low even between the members of same FAE family. As expected, when structural alignment was performed between different FAE family members, an average of only 37 residues were structurally aligned, raising the possibility of considering FAEs as ‘non-homologous isofunctional enzyme superfamily’. These observations indicate that despite of low sequence similarity, a certain extent of structural homology is preserved within each FAE family. It is also well known that enzymes with the same fold catalyze the same reaction even in absence of significant sequence similarity [50].

**Table 2:** Cross-structure statistics for 3D structure alignment of FEF family members of *A.oryzae* feruloylome.

Cross-structure statistics: RMSD^1^						Cross-structure statistics: Sequence Identity^2^					
FEF	Structure	A.O.1	A.O.2	A.O.3	A.O.11	FEF	Structure	A.O.1	A.O.2	A.O.3	A.O.11
FEF 4	**A.O.1**		1.862	3.755	3.893	FEF 4	**A.O.1**		0.396	0.123	0.123
	**A.O.2**	1.862		3.548	3.694		**A.O.2**	0.396		0.160	0.144
	**A.O.3**	3.755	3.548		2.707		**A.O.3**	0.123	0.160		0.299
	**A.O.11**	3.893	3.694	2.707			**A.O.11**	0.123	0.144	0.299	
		**A.O.4**	**A.O.5**					**A.O.4**	**A.O.5**		
FEF 6	**A.O.4**		3.380			FEF 6	**A.O.4**		0.142		
	**A.O.5**	3.380					**A.O.5**	0.142			
		**A.O.6**	**A.O.7**	**A.O.8**	**A.O.9**			**A.O.6**	**A.O.7**	**A.O.8**	**A.O.9**
FEF 7	**A.O.6**		3.077	3.034	3.369	FEF 7	**A.O.6**		0.229	0.193	0.188
	**A.O.7**	3.077		3.388	3.722		**A.O.7**	0.229		0.177	0.234
	**A.O.8**	3.034	3.388		1.887		**A.O.8**	0.193	0.177		0.323
	**A.O.9**	3.369	3.722	1.887			**A.O.9**	0.188	0.234	0.323	
		**A.O.10**	**A.O.13**					**A.O.10**	**A.O.13**		
FEF 12	**A.O.10**		3.583			FEF 12	**A.O.10**		0.191		
	**A.O.13**	3.583					**A.O.13**	0.191			

1 RMSD stands for the Root Mean Square Deviation, calculated between Cα-atoms of matched residues at best 3D superposition of the query and target structures. RMSD is presented in angstroms. In simple words, RMSD gives you an idea how separated, at best 3D superposition, a “typical” pair of matched Ca-atoms is.

2 Sequence identity is a quality characteristic of Cα-alignment. It is calculated from structure (3D), rather than sequence alignment. Therefore, two almost identical sequences may be estimated at low sequence identity if they fold into slightly different structures.

Secondary structure alignments can often show relationships between the proteins that are not immediately obvious from sequence identity alone. For example, the results from the alignments of Secondary Structure Elements (SSEs) showed that A.O.1 and A.O.2 that fall under FEF 4 share 33% sequence identity and surprisingly 88% SSE similarity. Figure 1 shows the regions of similar SSEs among different feruloyl esterase family members. As expected, proteins from different FAE families showed low sequence identity and SSE similarity. For example, A.O.1 from FEF 4 (Figure 1A) and A.O.4 that falls under FEF 6 (Figure 1B) share 9% sequence identity and 19% SSE similarity.

Given that the members within a certain FAE family have high degree of SSE similarity, the question we posed was if they also share similar configuration of binding sites. This was investigated by analyzing the residue-by-residue 3D mapping of the structures at their best superimposition states. The residue-by-residue 3D mapping and 3D structural alignment depicted in ribbon structures for the *A. oryzae* members of the FEF 4, FEF 6, FEF 7 and FEF 12 families are given in Supporting Information S3, S4, S5 and S6, respectively. The rotation-translation matrices of the best superposition of the respective members for the FAE families are also given in the Supporting Information files mentioned above.

The residue-by-residue 3D mapping in the regions of similar secondary structure folds around the binding pockets indicates that the proteins within a particular FAE family adopt similar binding environment. It is intriguing that the pattern of residues within the binding pocket of FAEs belonging to different families is very diverse. This highlights the importance of the pre-binding dynamics in the substrate recognition. Therefore, we suggest that the varied substrate specificity of the proteins among the FAE families is dictated by their overall structural fold in the binding pocket regions rather than the complete sequence identity.

To further investigate and confirm the structural characteristics we defined *in silico*, we subsequently cloned and expressed three *A. oryzae* FAEs belonging to three different FAE families and analyzed them by defining their substrate profiling and changes in structure as a function of pH were probed using CD spectroscopy.

### Molecular Cloning and Expression of A.O.2, A.O.8 & A.O.10 FAEs

The genes of the three FAEs of the *A. oryzae* feruloylome designated as A.O.2, A.O.8 and A.O.10 that fall under different FAE families, FEF 4 (sub-family A), FEF 7 (sub-family A) and FEF 12 (sub-family B), respectively, were obtained via cDNA synthesis from *A. oryzae* total RNA, cloned into pPICZα-C vector and expressed in *P. pastoris*. The observed molecular masses (MM) of the purified (Ni^2+^ -affinity chromatography) A.O.2, A.O.8 and A.O.10 on SDS-PAGE were in accordance to the computationally estimated values ∼64 kDa, ∼60 kDa and ∼65 kDa, respectively. Note that the C-terminal tag will add 2.5 kDa to the size of the protein and it was therefore included in the MM estimations.

### Substrate Spectra and Common Feature-based Pharmacophore (CFBP) Models for New FAE Families

In our previous study on developing the new FAE classification system [4], pharmacophore models were proposed for three sub-families based on key features of the substrate activity spectra of the respective members. Using the same procedures described in that study, we developed here pharmacophore models for two more FAE sub-families viz., FEF 7A and FEF 12B. In order to obtain the substrate spectra, the three recombinant FAEs were experimentally tested for activity against a series of 15 methyl cinnamate esters (Table 3). The predicted substrate specificity profile for the A.O.2 based on the pharmacophore model that we previously developed for the FEF 4 using literature data (7) shows ∼95% accuracy to the experimental characterization that was performed in this study. The remaining two FAEs viz., A.O.8 and A.O.10 that belong to the FEF 7 and FEF 12 families, respectively, showed a novel pattern of substrate specificity profile as shown in Table 3. Common feature pharmacophore models based on the observed substrate spectra were generated for A.O.8 and A.O.10 using the HipHop algorithm [4], [60]. Both active and inactive substrates for the respective enzymes were given as input. We defined that the active substrates of the respective enzyme must map completely or partially to the pharmacophore, while the features from the inactive substrates (on which the respective enzyme has no observed activity) must be considered as “NOT” features. The top ranked pharmacophores for A.O.8 and A.O.10 are presented in Figure 2A and 2B. None of the three inactive substrates of A.O.8 align with the Hydrogen Bond Acceptor (HBA) features of the pharmacophore model (Figure 2C). The same is the case for the inactive substrates of the A.O.10 (Figure 2D). Figure 2E shows the comparison of both pharmacophore models, and the ‘sensitive’ HBA3 feature (Figure 2B) that forms the basis of slightly different substrate spectra between A.O.8 and A.O.10. Therefore, the developed pharmacophore models could be applied for virtual screening of compound databases for short listing the putative substrates prior to docking studies or for post-processing docking results to remove false positives.

**Table 3:** Substrate specificity of characterized FAEs that belong to five different sub-families for the methyl esters of a series of 15 substituted cinnamic acids. *K_m_* is expressed as mM.

	FEF 12A	FEF 4B	FEF 4A	FEF 4A	FEF 7A	FEF 12B
	AnFAEA^1^	TsFAEC^2^	AnFAEB^1^	A.O.2	A.O.8	A.O.10
**MFA**	0.72	0.04	1.32	1.39	1.72	2.14
**MCA**	ND	<0.005	0.22	2.36	1.95	3.02
**MPC**	ND	0.01	0.014	1.51	ND	ND
**MSA**	0.45	0.37	ND	ND	7.06	6.44
**M2C**	ND	0.015	ND	1.73	3.99	1.64
**M3C**	ND	0.025	0.55	2.55	2.12	2.21
**MC**	ND	0.118	0.79	3.14	1.03	ND
**MTM**	1.63	ND	ND	ND	1.92	3.8
**M2M**	ND	ND	0.72	0.73	ND	0.67
**M3M**	1.99	0.301	ND	ND	1	0.75
**M4M**	ND	ND	0.31	0.55	3.48	ND
**M34DC**	1.36	0.626	ND	ND	ND	0.4
**M35DC**	0.92	0.075	ND	ND	4.75	4.06
**M34MC**	ND	0.533	0.85	1.47	2.57	1.77
**M43PP**	2.08	0.016	3.17	8.64	6.24	7.25

ND, Not Detected.

1 Values taken from Topakas *et al* [65].

2 Values taken from Vafiadi *et al* [66].

### Structure-function Relationship Analysis of Expressed FAEs

CD spectroscopy data were collected for the three expressed FAEs (A.O.2, A.O.8 and A.O.10) in the pH range 3.0–11.0 to gain insights of respective structural elements and identify under which conditions significantly structural changes occur. The curves obtained from CD spectroscopy for the respective recombinant enzymes are given in Figure 3. Graphs showing secondary structure content of the three proteins at different pH obtained through deconvolution of CD spectra and relative activity at respective pH are given in Figure S2. The CD spectroscopy curves for the A.O.2 protein showed similar pattern in the range pH 7.0–10.0 (Figure 3A) and the curves were distorted at extreme pH (pH ≤5.0 and pH 11.0) indicating unfolded or disordered protein. Deconvolution of A.O.2 CD spectra showed that more than 90% of the β-sheet content unfolds beyond pH 10. The reason for such rapid unfolding of the A.O.2 is the presence of large amount (≥35% of total structure at pH 7.0) of random coils and turns in its structure making the secondary structure elements more accessible by the surrounding environment. The pH-dependence of the enzyme activities (Figure 4A) of A.O.2 showed that the enzyme is active between pH 7.0–10.0 suggesting that the A.O.2 activity is conformation-dependent.

Interestingly, in the case of the A.O.8 protein, all the CD spectroscopy curves at different pH showed similar pattern (Figure 3B) and the deconvolution of data between 190–240 nm indicated that the content of α-helices and β-sheets were stable over a wide pH range and hence the enzyme may have a similar activity at these pH values. But, in contrary, A.O.8 showed pH optima at pH 9.0, and considerable high activity at pH 10.0 and pH 11.0, whereas enzyme activity below pH 7 was low (Figure 4B).

The A.O.10 CD spectra profile indicates the presence of three distinct groups of curves (Figure 3C) in three pH range viz., pH 3.0–5.0, pH 6.0–8.0, and pH 9.0–11.0. Deconvolution of A.O.10 CD spectra showed that the enzyme attains three different conformations in the above mentioned pH range. A.O.10 is characterized by high content of β-sheets within the structure between pH 3.0–5.0, while many β-sheets were unfolded and α-helix content was higher in the neutral pH range 6.0–8.0. In the third pH range (9.0–11.0), approximately 40% α-helices were unfolded making the protein inactive. Activities for the A.O.10 were observed in the second pH range, while no activity was observed in the other extremes (Figure 4C), suggesting the conformation dependent activity behavior for A.O.10.

### Identification of Active Site Residues Using Amino Acid Titration Curves: Insight into the Binding Pocket Microenvironments of Expressed FAEs

The analysis of titration curves of specific residues as a function of pH aids in the prediction of potential active site residues. A cluster of few residues with perturbed titration curves is a reliable predictor of active site location [61]. The identification of catalytic residues plays an important role in complementing the experimental characterization of the enzyme. The active site residues of A.O.2, A.O.8 and A.O.10 predicted by using protonation state of individual residues at different pH and the respective titration curves are shown in Figure 5. The predicted catalytic triad residues of A.O.2 are SER 183, ASP 419 and HIS 465. The predicted catalytic triad residues of A.O.8 are SER 177, ASP 401 and HIS 437. The predicted catalytic triad residues of A.O.10 are SER 177, ASP 437 and HIS 483. The catalytic triad residue predictions through simulations in the present study correlate and strengthen the catalytic triad residue predictions through evolutionary analysis of functionally important residues [62] in the descriptor-based classification system for feruloyl esterases that has been proposed recently [4]. The selection of the active site serine residue was based on the nucleophilic elbow pattern ‘G-X-S-X-G’ position of the respective protein (where G is glycine; X denotes ‘any’ amino acid; S is for Serine).

In FAEs that use the classical SER-HIS-ASP catalytic triad mechanism, serine acts as nucleophile, histidine as the general acid-base, and the aspartic acid helps to orient the histidine residue and further neutralize the charge that forms on histidine during the catalytic process [63]. So, the protonation states or the net charge of aspartic acid and histidine residues are very important for maintaining the activity of FAEs. We observed that ASP and HIS residues in all the three proteins have a strikingly different predicted titration function.

Protein forces that control the activity profile are not directly accessible by laboratory experiments. However, the protein function is significantly determined by the spatial structure. The modeled FAE structures enables us to inspect the structural framework of amino acids surrounding the catalytic triad residues that provides a basis for understanding the factors responsible for different titration curves of the active site residues among different FAEs. The screenshots of A.O.2, A.O.8 and A.O.10 binding pockets created using Discovery Studio software are given in Figure S3. Amino acid residues within the radius of 7 Å are shown in each of the binding pocket. The hydrogen bonding patterns of active site residues with the surrounding amino acids are depicted in red dashed lines. From the binding pocket microenvironments, it is evident that perturbed protonation states of the active site aspartic acid and histidine residues are regulated by the surrounding amino acids.

For example, in the case of A.O.10, the ASP437 carboxylate can be easily deprotonated due to the geometry and type of the surrounding amino acid residues (see Figure S3). The first oxygen (OD1) of ASP437 carboxylate is in close vicinity of four surrounding amino acid residues. OD1 is potentially involved in hydrogen bonding with the NH groups of ILE 440, SER 439, SER 454 and γ-H of SER 454; OD2 of the ASP437 carboxylate is also involved in a hydrogen bond with NH group of ILE 440.

In the case of the A.O.8, the partial protonation state of ASP 401 can be explained by the fact that only one of the oxygen (OD2) belonging to the carboxylate group is involved in hydrogen bonding with the NH groups of the two hydrophobic amino acids viz., MET 399 & VAL 403, whereas oxygen (OD1) might be involved in the buffering of HIS 437 during the catalytic mechanism (see Figure S3). Unlike the active site histidine group of A.O.2 and A.O.10, the histidine (HIS 437) of A.O.8 is not involved in hydrogen bonding with the surrounding amino acids. In highly polarizable binding pocket microenvironments, the charged form of the catalytic residue (ASP or HIS) ionizable group will predominate. The presence of A.O.8 catalytic residues in such microenvironment makes their charges more stable without the protein undergoing any significant structural reorganization. On the other hand, the less polarizable binding pocket microenvironments leads to the neutral form of catalytic residue (ASP or HIS) with concomitant shift in pK values [64]. The extent of hydrogen-bonding interactions by surrounding residues will have more effect on ASP compared to HIS, due to the higher hydrogen bonding potential of the ASP carboxylic group than the HIS amino group. It should not be ignored that the protonation states of internal ionizable groups are also sensitive to protein conformation. Hence, it can be explained that the cooperative effect of hydrogen bond-interactions by surrounding amino acid residues of the binding pocket can dictate the protonation states of the active site residues of the respective enzyme. No ionizable atoms were found in half of the substrates used in this study. The remaining substrates do not possess any ionizable atoms near the ester bond where the nucleophilic attack/catalysis takes place. Microspecies distribution diagrams at different pH for the substrates used in this study are given in Supporting Information S7.

The combined observations of hydrogen-bonding interactions provide steering clues for rational enzyme engineering via substitution of targeted amino acid residues without triggering any major conformational reorganization. A small number of critical mutations affecting the protonation states of ASP or HIS catalytic residues may be sufficient to fine-tune the activity of FAEs. For example, substitution of residues in binding pocket with highly polar amino acid residues could change the protonation states of the active site residues enabling the proteins to withstand and remain active at extreme pH range.

### Conclusion

From the analysis of the modeled FAE structures of *A. oryzae* feruloylome, high structural similarity of SSEs was observed between the members that belong to the same FAE family, but there was no consensus on the structural features that contribute to the substrate specificity between different FAE family members. The differences in substrate specificity profiles of FAE families are not surprising in view of the low structural similarity between the families. The modeled structures shows that, with a limited set of structural scaffolds, FAEs evolved into different families and further analysis of binding pockets indicated the topological variations of FAEs that leads to a wide spectrum of substrate specificities. A detailed understanding of protein structural elements of each FAE family that define the substrate specificity and other functional characteristics like pH dependence is clearly of utmost importance for designing and predicting substrate-enzyme binding and was extensively studied here.

This is to the best of our knowledge the first time that the active site residues of FAEs were probed using molecular dynamics simulations. 3D mapping of enzyme binding pockets revealed the microenvironment of amino acid residues involved in the inter-connection between the structure and different substrate specificity profiles. Such observations of the binding pocket microenvironments will guide the engineering of enzymes tolerant to the extreme reaction conditions. Future structural studies with different cognate ligand-receptor complexes using X-ray crystallography/NMR complemented with analysis of cognate ligand-mutated receptor complexes will further extend our understanding of characteristic fingerprints that guide the varied substrate specificities among the members of different FAE families.

## Supporting Information

Figure S1 Solvent surface rendered structures of the modeled FAEs with high C-Score colored according to the secondary structure (Red indicates beta sheet regions; Cyan indicates alpha helix regions; White indicates connecting loops and strands). The model structures and their corresponding PMDB accession code are (A) A.O.1 with accession code PM0077341. (B) A.O.2 with accession code PM0077342. (C) A.O.3 with accession code PM0077343. (D) A.O.4 with accession code PM0077344. (E) A.O.5 with accession code PM0077345. (F) A.O.6 with accession code PM0077346. (G) A.O.7 with accession code PM0077347. (H) A.O.8 with accession code PM0077348. (I) A.O.9 with accession code PM0077349. (J) A.O.10 with accession code PM0077350. (K) A.O.11 with accession code PM0077351. (L) A.O.12 with accession code PM0077352. (M) A.O.13 model with accession code PM0077353.
(DOC)

Figure S2 Binding pocket environments of expressed FAEs. The catalytic triad residues are shown in magenta, where as the residues within the radius of 7 Å are colored in green. Hydrogen-bonding interactions are shown as red dashed lines. (A) A.O.2. (B) A.O.8. (C) A.O.10.
(DOC)

Figure S3 Binding pocket environments of expressed FAEs. The catalytic triad residues are shown in magenta, where as the residues within the radius of 7 Å are colored in green. Hydrogen-bonding interactions are shown as red dashed lines. (A) A.O.2. (B) A.O.8. (C) A.O.10.
(DOC)

Table S1 Physico-chemical parameters and post-translational modifications for feruloylome of *Aspergillus oryzae.*
(DOC)

Table S2 Primers used for cDNA synthesis. Few nucleotides (shown in magenta) were also added to be in the reading frame of the pPICZα-C vector after analyzing the vector constructs with the Clone Manager software.
(DOC)

Table S3 Structure evaluation scores for modeled FAEs of *A. oryzae* feruloylome.
(DOC)

Supporting Information S1 Feruloylome of *Aspergillus oryzae.*
(XLSX)

Supporting Information S2 PDB structures used for multi template modeling and their secondary structure alignments.
(XLSX)

Supporting Information S3 3D structural alignment for FEF 4 members of A. oryzae feruloylome.
(XLSX)

Supporting Information S4 3D structural alignment for FEF 6 members of A. oryzae feruloylome.
(XLSX)

Supporting Information S5 3D structural alignment for FEF 7 members of A. oryzae feruloylome.
(XLSX)

Supporting Information S6 3D structural alignment for FEF 12 members of A. oryzae feruloylome.
(XLSX)

Supporting Information S7 Microspecies distribution diagrams at different pH for the ionizable substrates.
(DOC)
