# Supplementary material for: Common and Distant Structural Characteristics of Feruloyl Esterase Families from Aspergillus oryzae
Source: PLoS One. 2012 Jun 22;7(6):e39473. doi: 10.1371/journal.pone.0039473 (PMC3382194; doi:10.1371/journal.pone.0039473)

**Figure S1.** Solvent surface rendered structures of the modeled FAEs with high C-Score colored according to the secondary structure (Red represent alpha helix regions; Cyan represent beta strand regions; Green represent coil regions). The model structures and their corresponding PMDB accession code are (*A*) A.O.1 with accession code PM0077341. (*B*) A.O.2 with accession code PM0077342. (*C*) A.O.3 with accession code PM0077343. (*D*) A.O.4 with accession code PM0077344. (*E*) A.O.5 with accession code PM0077345. (*F*) A.O.6 with accession code PM0077346. (*G*) A.O.7 with accession code PM0077347. (*H*) A.O.8 with accession code PM0077348. (*I*) A.O.9 with accession code PM0077349. (*J*) A.O.10 with accession code PM0077350. (*K*) A.O.11 with accession code PM0077351. (*L*) A.O.12 with accession code PM0077352. (*M*) A.O.13 model with accession code PM0077353.

(A)

(B)

(C)

(D)


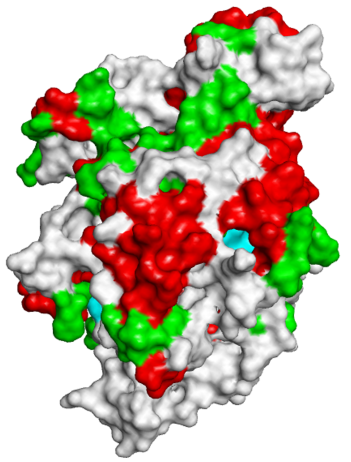

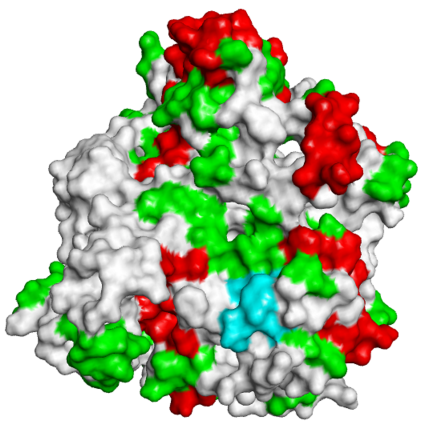

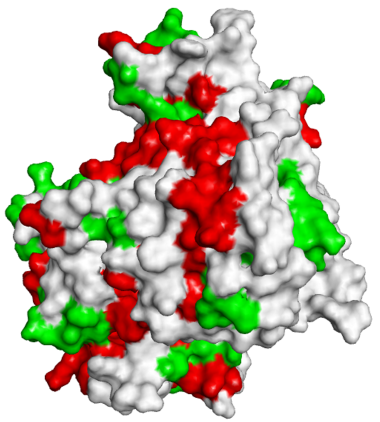

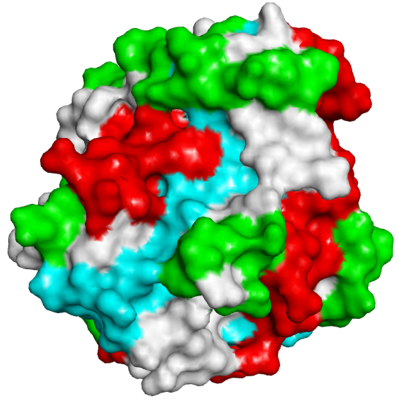


(E)

(F)

(G)

(H)


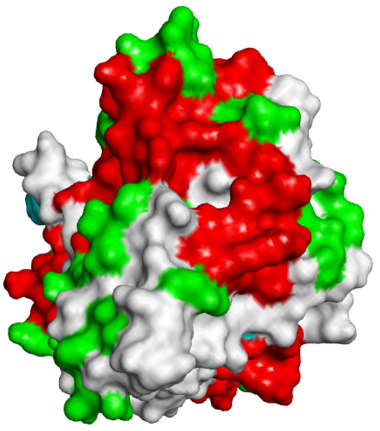

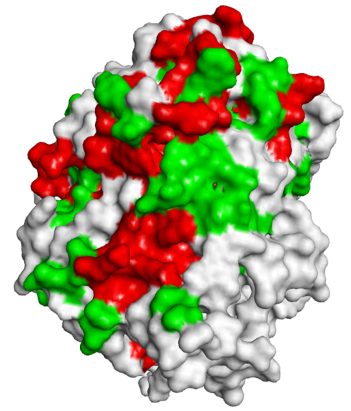

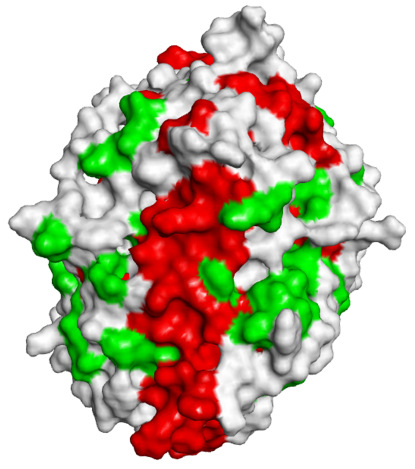

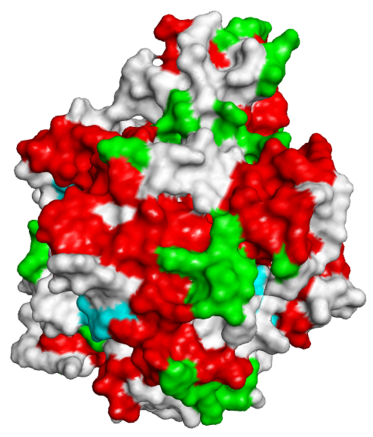


(I)

(J)

(K)

(L)


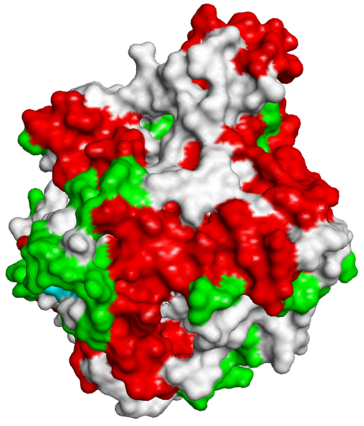

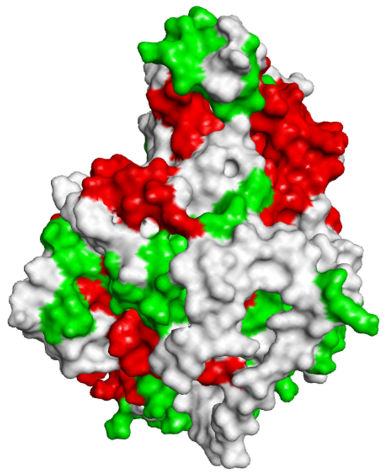

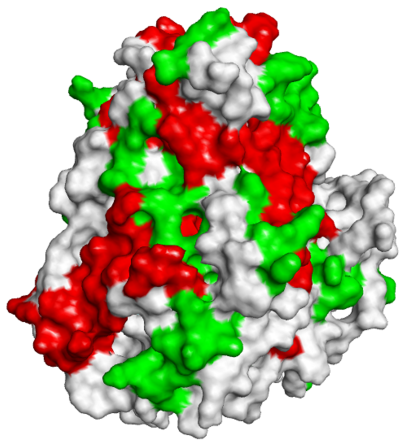

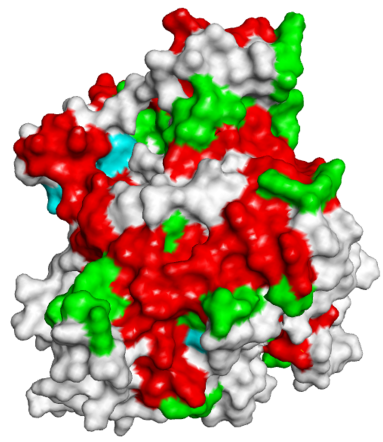


(M)


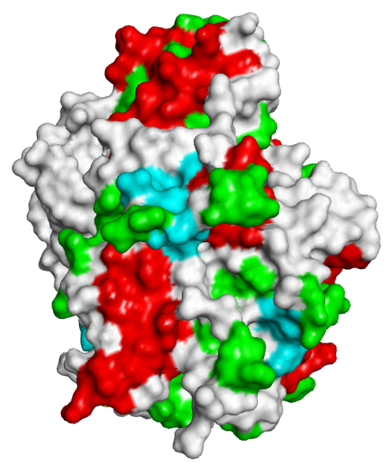

Supplement: Figure S1 — Solvent surface rendered structures of the modeled FAEs with high C-Score colored according to the secondary structure (Red indicates beta sheet regions; Cyan indicates alpha helix regions; White indicates connecting loops and strands). The model structures and their corresponding PMDB accession code are (A) A.O.1 with accession code PM0077341. (B) A.O.2 with accession code PM0077342. (C) A.O.3 with accession code PM0077343. (D) A.O.4 with accession code PM0077344. (E) A.O.5 with accession code PM0077345. (F) A.O.6 with accession code PM0077346. (G) A.O.7 with accession code PM0077347. (H) A.O.8 with accession code PM0077348. (I) A.O.9 with accession code PM0077349. (J) A.O.10 with accession code PM0077350. (K) A.O.11 with accession code PM0077351. (L) A.O.12 with accession code PM0077352. (M) A.O.13 model with accession code PM0077353. (DOC) [file pone.0039473.s001.doc]
