# Supplementary material for: Common and Distant Structural Characteristics of Feruloyl Esterase Families from Aspergillus oryzae
Source: PLoS One. 2012 Jun 22;7(6):e39473. doi: 10.1371/journal.pone.0039473 (PMC3382194; doi:10.1371/journal.pone.0039473)
Supplement: Figure S2 — Binding pocket environments of expressed FAEs. The catalytic triad residues are shown in magenta, where as the residues within the radius of 7 Å are colored in green. Hydrogen-bonding interactions are shown as red dashed lines. (A) A.O.2. (B) A.O.8. (C) A.O.10. (DOC) [file pone.0039473.s002.doc]

**Figure S2.** Graphs showing secondary structure content of the three proteins at different pH obtained through deconvolution of CD spectra and relative activity at respective pH. (A) A.O.2. (B) A.O.8. (C) A.O.10.

**A**


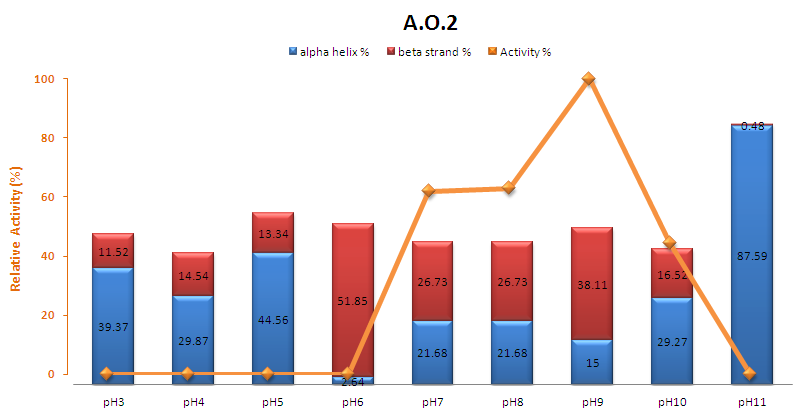


**B**

**
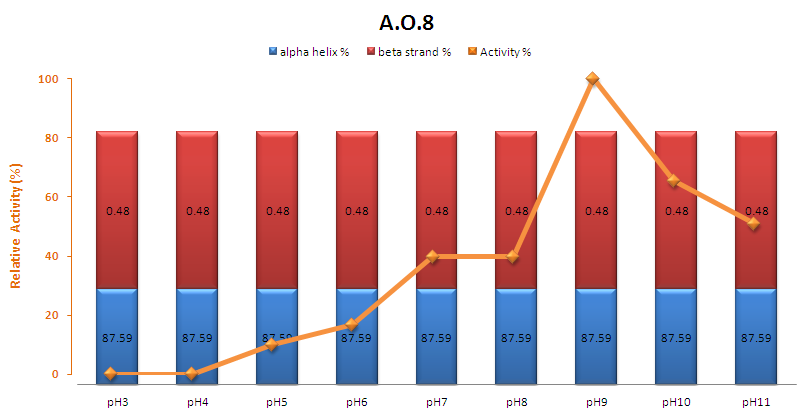
**

**C**

**
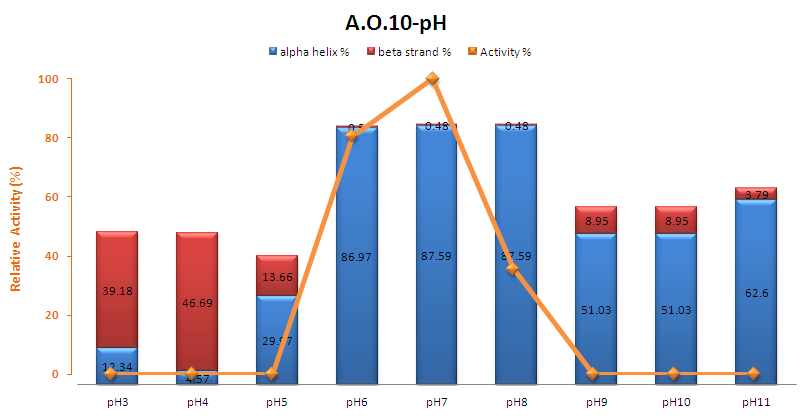
**
