# Supplementary material for: Common and Distant Structural Characteristics of Feruloyl Esterase Families from Aspergillus oryzae
Source: PLoS One. 2012 Jun 22;7(6):e39473. doi: 10.1371/journal.pone.0039473 (PMC3382194; doi:10.1371/journal.pone.0039473)
Supplement: Table S1 — Physico-chemical parameters and post-translational modifications for feruloylome of Aspergillus oryzae. (DOC) [file pone.0039473.s004.doc]

**Table S1.** Physico-chemical parameters and post-translational modifications for feruloylome of *Aspergillus oryzae*

| **ID** | **Length of Native Protein** | **Length of Signal Peptide** | **Length of Mature Protein** | **Molecular weight** | **Theoretical pI** | **Extinction coefficient^1^** | **Absorbance^2^** |
| --- | --- | --- | --- | --- | --- | --- | --- |
| A.O.1 | 446 | No | 446 | 49.98 kD | 4.8 | 115780 | 2.316 |
| A.O.2 | 573 | 1 to 19 | 554 | 61.35 kD | 4.98 | 112815 | 1.839 |
| A.O.3 | 525 | 1 to 19 | 506 | 55.71 kD | 4.93 | 89685 | 1.61 |
| A.O.4 | 281 | 1 to 21 | 260 | 28.1 kD | 4.61 | 47705 | 1.697 |
| A.O.5 | 307 | 1 to 19 | 288 | 31.19 kD | 4.89 | 71070 | 2.278 |
| A.O.6 | 516 | 1 to 17 | 499 | 55.08 kD | 4.66 | 116825 | 2.121 |
| A.O.7 | 514 | 1 to 18 | 496 | 53.82 kD | 5.04 | 99405 | 1.847 |
| A.O.8 | 524 | 1 to 21 | 503 | 54.64 kD | 5.56 | 97010 | 1.775 |
| A.O.9 | 502 | No | 502 | 54.19 kD | 5.15 | 96885 | 1.788 |
| A.O.10 | 588 | 1 to 18 | 570 | 62.28 kD | 4.65 | 119235 | 1.914 |
| A.O.11 | 526 | 1 to 19 | 507 | 55.80 kD | 5.43 | 87960 | 1.576 |
| A.O.12 | 529 | 1 to 37 | 492 | 53.08 kD | 4.85 | 88070 | 1.661 |
| A.O.13 | 540 | 1 to 18 | 522 | 56.57 kD | 4.99 | 97470 | 1.723 |
| ^1^ Extinction coefficients are in units of M^-1^ cm^-1^, at 280 nm measured in water. | | | | |  |  |  |
| ^2^ Absorbance 0.1% (=1 g/l) | | |  |  |  |  |  |
| The physico-chemical parameters were calculated for mature proteins without native signal peptide | | | | | |  |  |
