# Supplementary material for: Common and Distant Structural Characteristics of Feruloyl Esterase Families from Aspergillus oryzae
Source: PLoS One. 2012 Jun 22;7(6):e39473. doi: 10.1371/journal.pone.0039473 (PMC3382194; doi:10.1371/journal.pone.0039473)
Supplement: Table S2 — Primers used for cDNA synthesis. Few nucleotides (shown in magenta) were also added to be in the reading frame of the pPICZα-C vector after analyzing the vector constructs with the Clone Manager software. (DOC) [file pone.0039473.s005.doc]

**Table S2.** Primers used for cDNA synthesis. Few nucleotides (shown in magenta) were also added to be in the reading frame of the pPICZα-C vector after analyzing the vector constructs with the Clone Manager software.

| **Gene** | **Primer** | **Restriction site at 5' end** |
| --- | --- | --- |
| 5'AOX1 primer | 5'-GACTGGTTCCAATTGACAAGC-3' | - |
| 3'AOX1 primer | 5'-GCAAATGGCATTCTGACATCC-3' | - |
| A.O.2 - Forward Primer | 5'-ACGAATTCAGCTAGCCTTAGCGATGTTTGC-3' | EcoRI |
| A.O.2 - Reverse Primer | 5'-AACGGTACCAAGTATAGAGGAATGCGATAGGCATC-3' | KpnI |
| A.O.8 - Forward Primer | 5'-CAGAATTCATCCAGATGCTCGCCAGC-3' | EcoRI |
| A.O.8 - Reverse Primer | 5'-AGTCTAGACATTTGGCGCAGGTGAAGGA-3' | XbaI |
| A.O.10 - Forward Primer | 5'-AGATCGATAGCTTCTTTTACCGATGTGTGC-3' | ClaI |
| A.O.10 - Reverse Primer | 5'-CGTCTAGACAGTATACAGGGACCTTGAAGGCT-3' | XbaI |
