# Supplementary material for: Common and Distant Structural Characteristics of Feruloyl Esterase Families from Aspergillus oryzae
Source: PLoS One. 2012 Jun 22;7(6):e39473. doi: 10.1371/journal.pone.0039473 (PMC3382194; doi:10.1371/journal.pone.0039473)
Supplement: Table S3 — Structure evaluation scores for modeled FAEs of A. oryzae feruloylome. (DOC) [file pone.0039473.s006.doc]

**Table S3.** Structure evaluation scores for modeled FAEs of *A. oryzae* feruloylome.

| **Protein** | **DOPE Score** | **DOPE-HR Score** | **Verify Score** | **Verify Expected High Score** | **Verify Expected Low Score** | **Initial Potential Energy (kcal/mol)** | **Potential Energy (kcal/mol)** |
| --- | --- | --- | --- | --- | --- | --- | --- |
| A.O.1 | -42144 | -35726 | 187 | 203 | 91 | -15768 | -25866 |
| A.O.2 | -54726 | -44075 | 199 | 253 | 114 | -18692 | -35241 |
| A.O.3 | -51181 | -43254 | 190 | 231 | 104 | -17075 | -28557 |
| A.O.4 | -30435 | -27904 | 129 | 118 | 53 | -9737 | -14305 |
| A.O.5 | -28913 | -22381 | 125 | 131 | 58 | 6142 | -14979 |
| A.O.6 | -50964 | -41678 | 193 | 227 | 102 | -16144 | -28017 |
| A.O.7 | -50244 | -41620 | 194 | 226 | 101 | -16751 | -28387 |
| A.O.8 | -49709 | -43150 | 193 | 229 | 103 | 584 | -28793 |
| A.O.9 | -48945 | -41716 | 198 | 229 | 103 | 382 | -27495 |
| A.O.10 | -54730 | -44111 | 194 | 260 | 117 | -3362 | -33413 |
| A.O.11 | -49918 | -40161 | 196 | 231 | 104 | -16969 | -29648 |
| A.O.12 | -49526 | -42203 | 195 | 224 | 101 | -16328 | -27236 |
| A.O.13 | -50518 | -41563 | 196 | 238 | 107 | -17632 | -30336 |
