# Supplementary material for: Common and Distant Structural Characteristics of Feruloyl Esterase Families from Aspergillus oryzae
Source: PLoS One. 2012 Jun 22;7(6):e39473. doi: 10.1371/journal.pone.0039473 (PMC3382194; doi:10.1371/journal.pone.0039473)

| **No.** | **Substrate** | **Ionizable** |
| --- | --- | --- |
| 1 | Methyl cinnamate | No |
| 2 | Methyl 2-hydroxy cinnamate | Yes |
| 3 | Methyl 3-hydroxy cinnamate | Yes |
| 4 | Methyl 4-hydroxy cinnamate (Methyl p-coumarate) | Yes |
| 5 | Methyl 3,4-dihydroxy cinnamate (Methyl caffeate) | Yes |
| 6 | Methyl 2-methoxy cinnamate | No |
| 7 | Methyl 3-methoxy cinnamate | No |
| 8 | Methyl 4-methoxy cinnamate | No |
| 9 | Methyl 3,4-dimethoxy cinnamate | No |
| 10 | Methyl 3,5-dimethoxy cinnamate | No |
| 11 | Methyl 3,4,5-trimethoxy cinnamate | No |
| 12 | Methyl 4-hydroxy-3-methoxy cinnamate (Methyl ferulate) | Yes |
| 13 | Methyl 3-hydroxy-4-methoxy cinnamate | Yes |
| 14 | Methyl 4-hydroxy-3,5-dimethoxy cinnamate (Methyl sinapate) | Yes |
| 15 | Methyl 4-hydroxy-3-methoxy phenyl propionate | Yes |

**Microspecies distribution diagrams at different pH for the ionizable substrates mentioned in the above table are given below:**

Methyl 2-hydroxy cinnamate


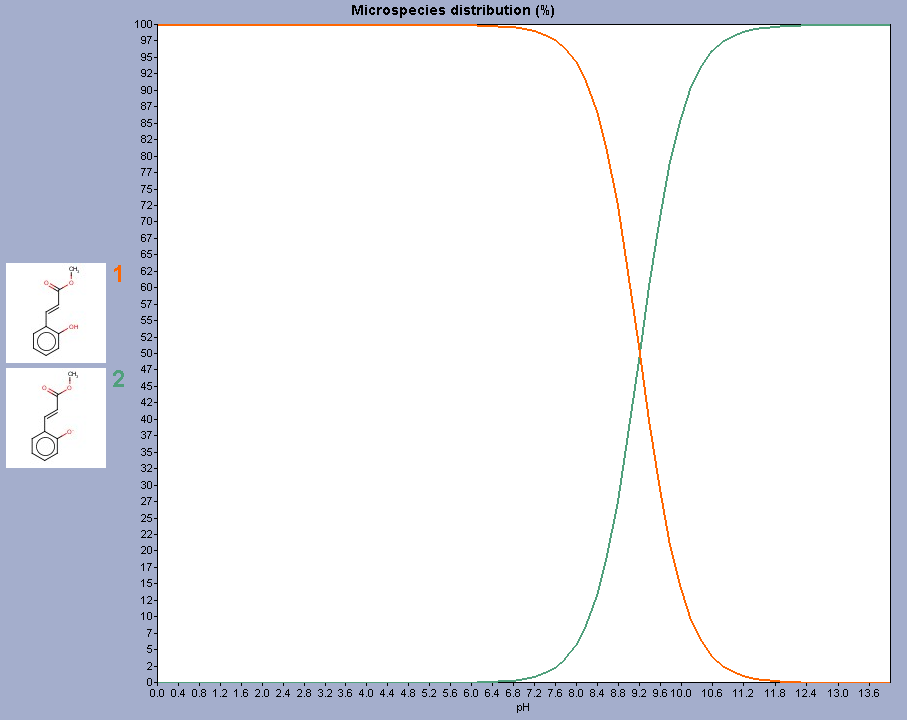


Methyl 3-hydroxy cinnamate


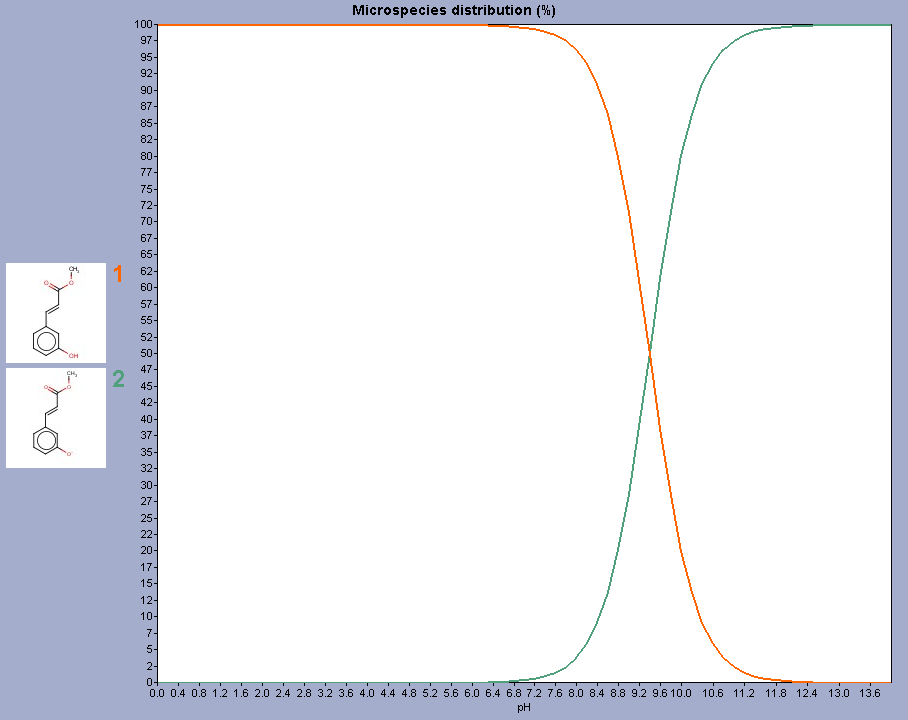


Methyl 4-hydroxy cinnamate (Methyl p-coumarate)


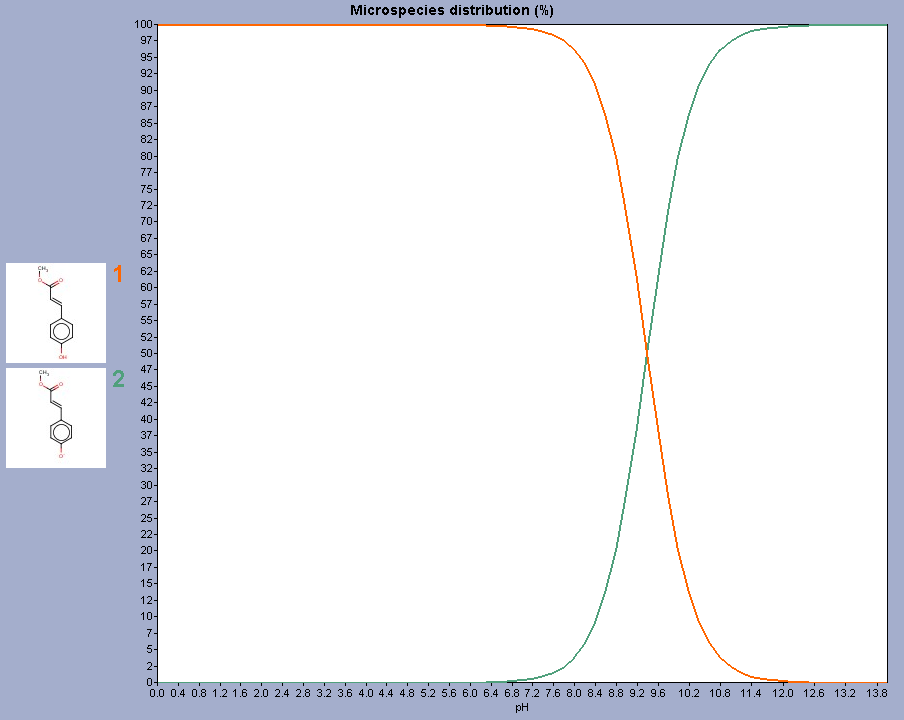


Methyl 3,4-dihydroxy cinnamate (Methyl caffeate)


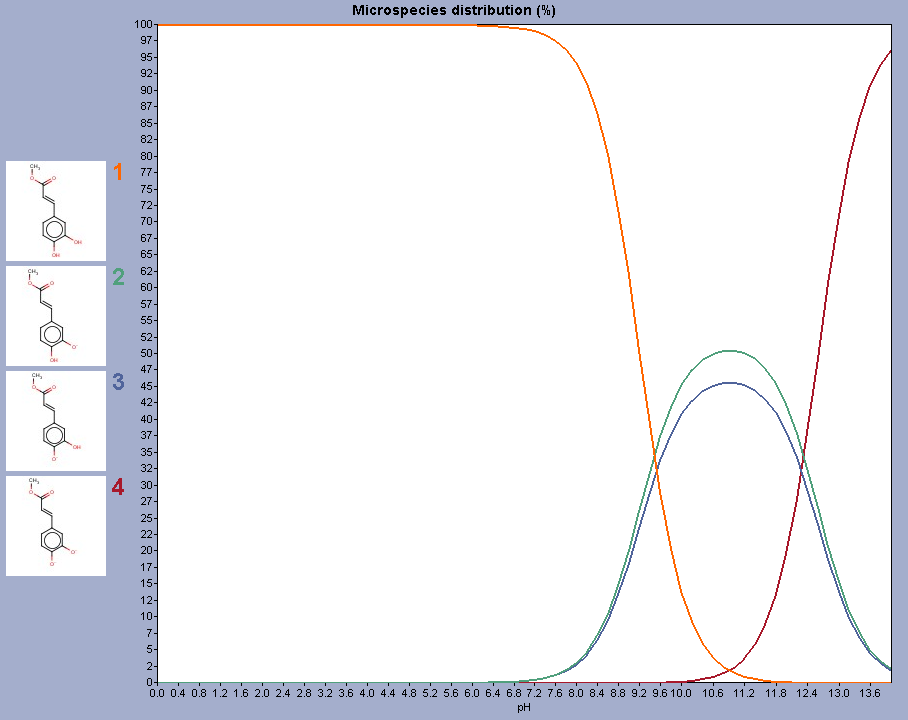


Methyl 4-hydroxy-3-methoxy cinnamate (Methyl ferulate)


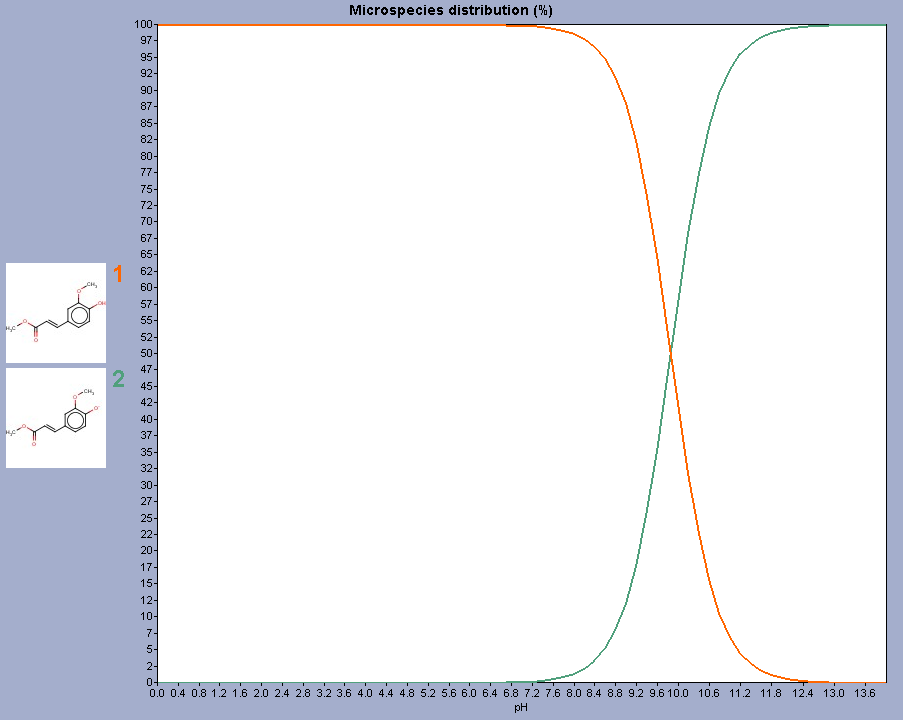


Methyl 3-hydroxy-4-methoxy cinnamate


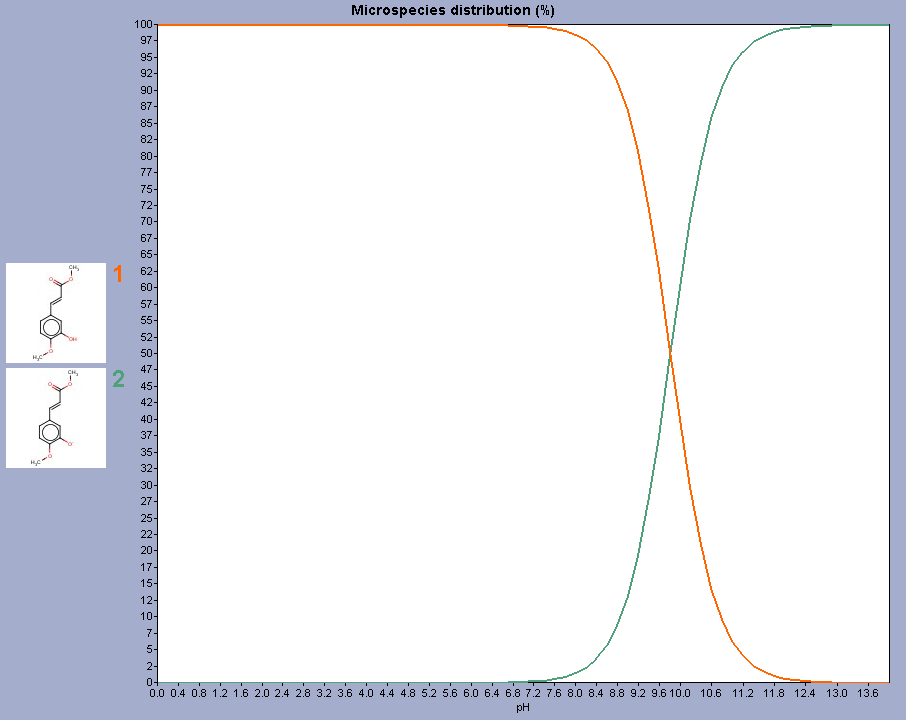


Methyl 4-hydroxy-3,5-dimethoxy cinnamate (Methyl sinapate)


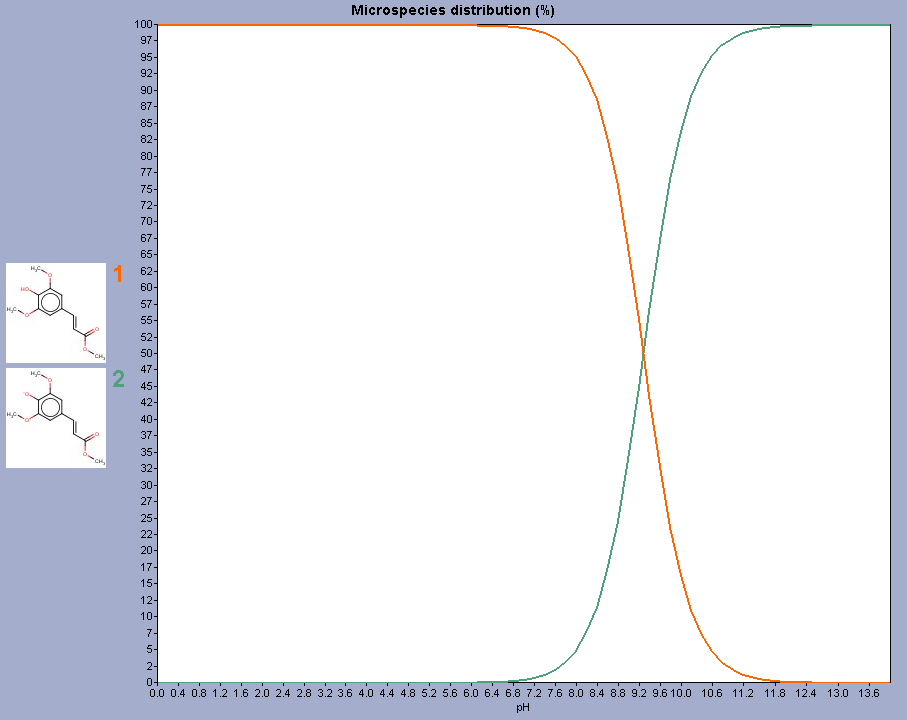


Methyl 4-hydroxy-3-methoxy phenyl propionate


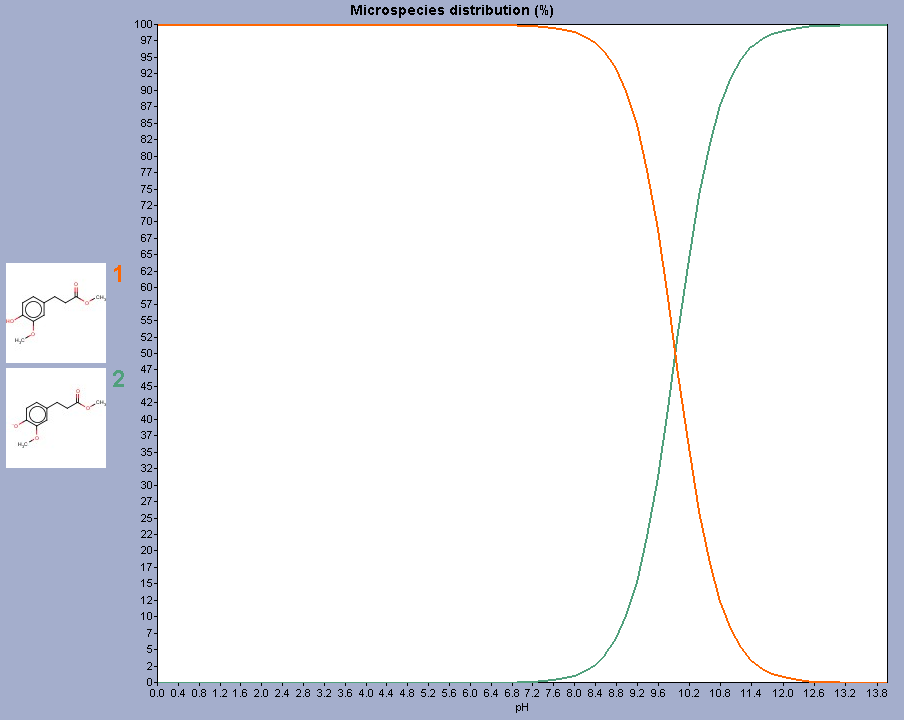

Supplement: Supporting Information S7 — Microspecies distribution diagrams at different pH for the ionizable substrates. (DOC) [file pone.0039473.s013.doc]
